# An infodemiology study on exploring the quality and reliability of colorectal cancer immunotherapy information

**DOI:** 10.1177/20552076231205286

**Published:** 2023-10-04

**Authors:** Hind Mohamed, Laura O’Malley, Dervla Kelly

**Affiliations:** 1 Master of Public Health Programme, School of Medicine, 8808 University of Limerick, Limerick, Ireland; 2 ULCaN and HIST research clusters, Health Research Institute, 8808 University of limerick, Limerick, Ireland

**Keywords:** Colorectal cancer immunotherapy, colorectal cancer immunotherapy treatment, colorectal cancer immunotherapy side effects, bowel cancer immunotherapy outcomes

## Abstract

**Background:**

Immunotherapy is a new treatment modality which promises hope for advanced colorectal cancer patients. To our knowledge, no previous studies have evaluated the quality of patient information available on this topic online.

**Objective:**

This study will explore the quality and reliability of colorectal cancer immunotherapy information using the *Journal of the American Medical Association* (JAMA) and DISCERN tools.

**Methods:**

Design thinking methodology was integrated with systematic scoping reviews framework to inform our descriptive observational media analysis study. Google Chrome was used to run four searches using prespecified search terms selected according to the top patient concerns about immunotherapy. The first 20 relevant results were identified (n = 80) and then duplicates were removed. Descriptive narrative univariate and bivariate analysis was done for the relevant variables.

**Results:**

The total of included websites was 17. Most websites score <3 points on JAMA and fair/poor on DISCERN. Most of the websites that score ≥3 points on JAMA and excellent/good on DISCERN have a charity affiliation. A total of 58.8% of the websites present the date, 41.2% demonstrate authorship, and sources are mentioned in 29.4% of the websites. Lack of content was noticed in providing the prognosis of patients if no treatment is given, clear aim and the effect of treatments on patient's quality of life.

**Conclusion:**

Assessing the reliability of information about cancer treatments online remains a challenge. Further research is required to understand the patient perceptions and use of online information and whether it has an impact on their behavioural health outcomes.

## Introduction

Colorectal cancer is the second most common cancer in women and the third in men.^1^ In Ireland, colorectal cancer is the second commonly diagnosed and fatal cancer as there are 2400 new cases detected annually contributing to 11% of cancer deaths.^2^ The atypical clinical presentation of colorectal cancer symptoms in the early stages of the disease along with underuse of screening tests leads to delay in its diagnosis and treatment.^3^

Colorectal cancer therapy is a comprehensive multidisciplinary process. It includes surgery, physical (radiotherapy), chemical (chemotherapy), biological (immunotherapy) and psychological therapy. Although surgery succeeds to clear the tumour in 80% of patients, 50% have micro-metastasis at the time of surgery. Chemotherapy is approved as an effective treatment modality for local metastasis but shows only modest efficiency against distant metastasis. Patients with distant metastasis have a 12% survival rate, and immunotherapy offers hope to patients with metastatic colorectal cancer.^4^

A distinguishing feature of metastatic colorectal cancer is its microsatellite stability status, which a biopsy measures. It can present as microsatellite stable, microsatellite instability-low and microsatellite instability-high and is used to match patients to immunotherapies.^3,5^

Immune checkpoint inhibitors immunotherapies specifically with monoclonal antibodies show promising outcomes in patients with microsatellite instability-high colorectal cancer and include pembrolizumab, nivolumab and ipilimumab.^6^ Beyond monoclonal antibodies, monospecific and bispecific antibodies, cellular therapies, vaccines and cytokines targeting other immune checkpoint molecules, macrophages and other components of innate immunity are under clinical trials for colorectal cancer patients.^3^

During the era of patient-centred care, cancer patients are becoming active participants in treatment-related decisions and information seeking has been demonstrated to play a critical role in helping patients cope with the uncertainties associated with cancer diagnosis and navigating treatment.^7^ A study done by Chua et al.^8^ (2018) found that cancer patients rely on the internet for obtaining information, focusing their search mainly on cancer diagnosis and treatment (90%), then side effects of treatments (87%), followed by complementary and alternative therapy (70%), depending on several factors.

A lot of studies on information seeking in cancer patients have been done and identified that age, gender, educational status, cancer type and stage of cancer at diagnosis play an important role in determining information seeking behaviour.^8–12^ It has been established in a study done in Singapore that female patients, those of a higher education standard and younger patients prefer to search for information online.^8^ Health status has also been found to be a significant factor in online health seeking.^8,13^ Houston and Allison^14^ reported that those who disclosed poor health status were more frequent users of the internet for health information. Internet users with chronic illnesses and comorbidities were noticeably more likely to use the internet as a source of information on health.^13^ Colorectal cancer patients were reported as less information seekers than patients with breast and prostate cancer, and they had concerns regarding credibility of the information available online.^9,15^

A study done by Fogg et al.^16^ (2003) explored that most consumers of web-based information depend on superficial and quick methods for credibility evaluation. They rely on site presentation and visual design rather than considering content and source presentation.^17^ This was also explained by Flanagin and Metzger as they found that web users try to use less mental efforts to judge reliability of online information. Finding high-quality sites is challenging, and there are problems with author identification, completeness and accuracy of the information provided on treatments.^18^

With the bombardment of pharmaceutical advertisements and media coverage, in conjunction with the undesirable side effects of treatments, understanding website quality is useful to inform educational initiatives to improve patient's digital literacy and use of online information during the cancer journey.^7^ Also, as immunotherapy is relatively new, no previous studies have evaluated the quality of information available on this topic for patients online. Thus, the health belief model was applied in our investigation as a concept to explain how individual behaviours can affect seeking and adherence to treatments and impact their physical health outcomes.^19^

## Aim and objectives

The aim of our study is to evaluate the quality and reliability of colorectal cancer immunotherapy information on patient-oriented websites using the *Journal of American Medical Association* (JAMA) benchmark and DISCERN tools. The objectives are as follows:

1. Categorise the quality of website's information using JAMA and DISCERN tools and describe each category by website's affiliation type.
2. Describe the website's information reliability by reporting their authorship, attribution of references, currency of the date content and disclosure/ownership of websites.
3. Describe the website's information reliability by reporting their aim, source and date of the information, knowledge uncertainty and bias of the information provided and how good the quality of information about treatment choices.

## Methodology

A human-centred, design thinking methodology approach was used in our study, and it includes the following steps in order: empathy, problem definition, ideation and designing a prototype and test its applicability.^20^ The first two steps were selected for application in our investigation to identify issues related to online information about immunotherapy from a patient perspective. The empathy stage was applied when conducting the search strategy, and problems were identified during the analysis and presented as key findings. This methodology was integrated with Arksey and O’Malley’s (2005) methodological framework of systematic scoping review for conducting our descriptive observational media analysis study.^21^

### Identify research question

The Population Concept and Context framework was used to develop the research question.^21^ The population consists of colorectal cancer patients, their carer's and the public. The concept is the quality and reliability of colorectal cancer immunotherapy information. The context is the online patient-oriented websites. So, the research question is ‘What is the quality and reliability of colorectal cancer immunotherapy information on patient-oriented websites?’

### Search strategy

**Figure 1.**
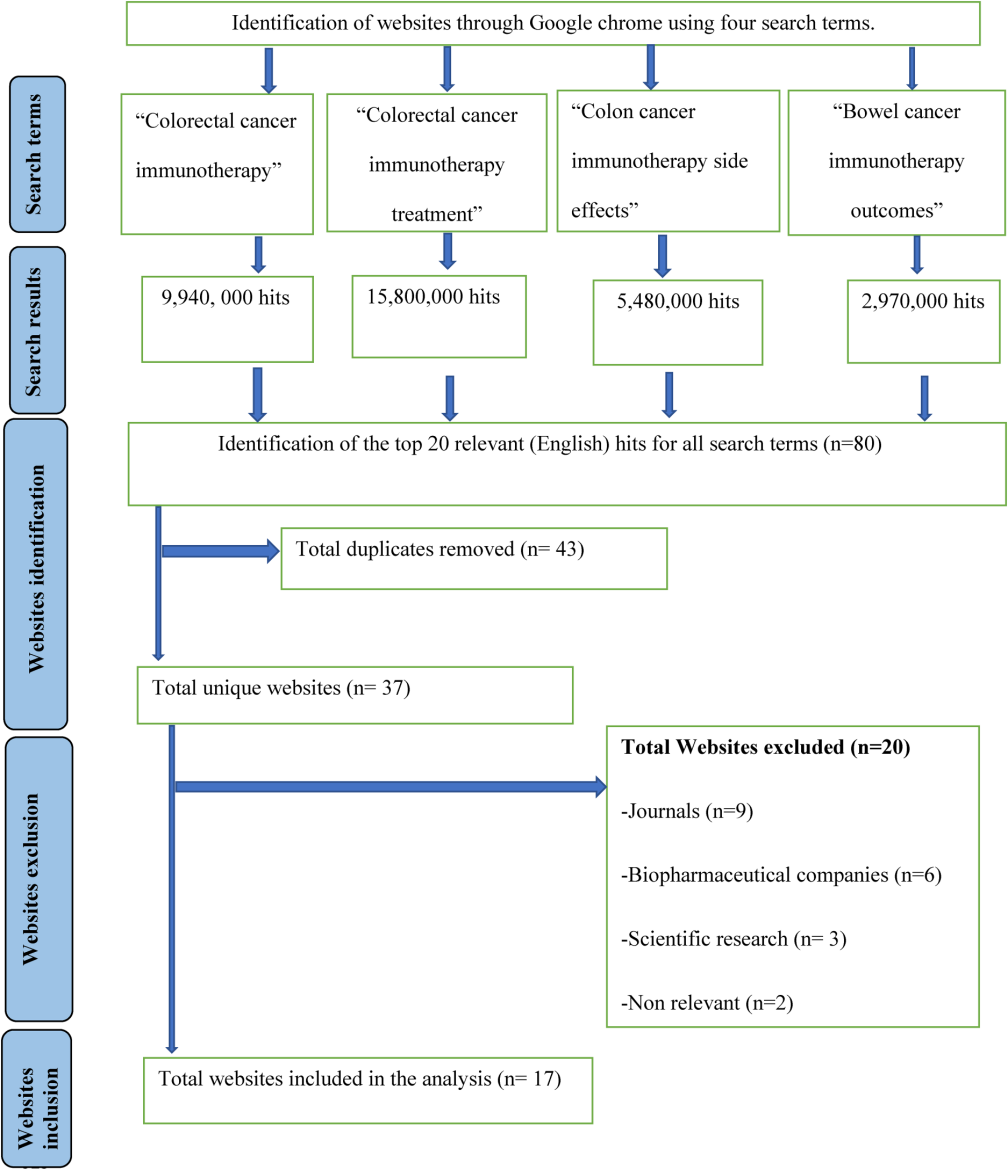
Consort flow diagram of website's inclusion and exclusion for evaluation.

A literature review research technique was used to understand the context of immunotherapy and to have an insight of user's concerns,^22^ barriers^15^ and needs^23^ (Figure 1, Appendix I). Search terms were selected according to the top patient concerns about immunotherapy.^22^

On 17 July 2021 at 9:00 am ECT, a clean-installed Google web browser was used to conduct the search using different search terms^24^ namely, ‘colorectal cancer immunotherapy”, ‘colorectal cancer immunotherapy treatment’, ‘colon cancer immunotherapy side effects’ and ‘bowel cancer immunotherapy outcomes’. The incognito window on Google Chrome was used to conduct the search, and the browser was cleaned to reduce the effect of Google search on personalised search algorithms.^25^ Clearance of browser history, cache and cookies was done to make sure that previous searches do not affect our search results.^26^ Google search engine was selected as it is the absolute traffic leader and commonly used engine by the public.^18^ Yahoo and Bing web browsers were screened after searching Google. The search conducted on Google identifies most of the links incorporated using Yahoo and Bing which are also commonly used browsers by internet users. This finding was also discovered by Mac et al.^26^ in a study done in Australia.

Previous research indicates that 90% of internet users view the first or second result pages (first 20 sites).^27^ So, the first 20 relevant (English) results were selected for each search term independently (n = 80). Duplicates (repeated appearance) were removed and noted for each search term and then across all search terms (n = 43). Websites were excluded (n = 20) if they are journals for healthcare providers (articles and books) (n = 9), commercial (biopharmaceutical companies) (n = 6), scientific research (n = 3) and non-relevant (other types of cancer or one paragraph provided) (n = 2). Websites were included if they provide information on immunotherapy for patients and their carers (n = 17) (Figure 1).

### Data extraction

Data was extracted for the included websites on an Excel sheet by one author, for the following variables: website's country and affiliation category, scope of immunotherapy information, types of immunotherapy treatment and all the JAMA and DISCERN variables. Noteworthy, 80% of the public are looking for a quality seal on websites displaying medical information.^28^ Therefore, data were also extracted on whether the Health On the Net Foundation (HON) code (HONcode), Patient Information Forum (PIF) TICK and Better Business Bureau (BBB) seals were displayed on the website page.^26,28,29^ The process of data extraction was done in 5 consecutive days, starting from 20 July 2021 to 24 July 2021.

Two investigators independently evaluated the quality of websites using the JAMA and DISCERN instruments; discrepancies were discussed until we reach a consensus. As JAMA has very broad definition for its criteria, the two investigators agree on the same definitions for the presence of each JAMA criteria. Variation was found between the investigator's DISCERN score, so the average (mean) total score for each website was taken.^27^

### Data analysis

Descriptive narrative univariate analysis was done for all the variables including as follows: website's country and affiliation category, the scope of immunotherapy information and types of treatment discussed and all the JAMA benchmark and DISCERN variables. Narrative bivariate analysis was done to describe the relationship between the JAMA and DISCERN score and website's affiliation category.

## Data quality tools

### JAMA benchmark

JAMA benchmark has four criteria to assess the quality of websites, including authorship, attribution, currency and disclosure. The presence of each criterion gives a score of one point.^26^

**Table 1.** JAMA criteria description, investigator's definition and number of websites adhering to investigator's definition with their specification.

Criteria ^26,31^	Criteria description ^26,31^	Investigator's definition for presence of each criterion	Number of websites n (%) adhering to investigator's definition (total n = 17)	Number of websites n (%) with specification of investigators definition for each criterion (total n = 17)
Authorship	Clearly identifiable author and contributors with affiliations and relevant credentials present.	Clearly identifiable one author or website's team author and/or reviewer with or without their affiliation and/or credential.	7 (41.2)	Website team author only n = 2 (11.8)
				One author + (one medical reviewer with credential) n = 1 (5.9)
				One author with credential n = 2 (11.8)
				One author without affiliation and credential n = 1 (5.9)
				One reviewer with affiliation and credential n = 1 (5.9)
Attribution	References and sources clearly listed with any copyright information disclosed.	Sources are mentioned at the end of the information provided or the webpage, either in a form of bibliography or links.	5 (29.4)	Bibliography n = 3 (17.6)
				Links n = 2 (11.8)
Disclosure	Website ownership clearly disclosed along with any sponsorship, advertising, underwriting and financial support.	Website ownership, sponsorship, advertising and sources of funding clearly declared.	17 (100)	All websites fully declare all specific criteria mentioned n = 17 (100)
Currency	Clearly identifiable posting date of any content as well as date of any revisions.	Copyright or review date is provided and/or date of sources.	10 (58.8)	Last and next review date with date of sources n = 1 (5.8)
				Last review date and date of sources with date of copyright n = 1 (5.8)
				Last review date and date of sources n = 2 (11.8)
				Last and next review date n = 1 (5.8)
				Date of copyright n = 5 (29.4)

### DISCERN

Discern instrument is a critical appraisal tool with a validated questionnaire for evaluation of written user health information.^27^ It is composed of 3 sections as follows: Section 1 (questions 1–8) evaluates the reliability of the information, whereas Section 2 (questions 9–15) assesses the information quality about treatment choices, and Section 3 (question 16) reflects an overall scoring rate independently of the previous 15 questions. Each question from the 16 has a ranging scale from 1 to 5, 1 indicates definite no and 5 definite yes. Partial scoring of 2–4 suggests the presence of some elements of the question.^30^ Although the DICERN tool has a very clear clarification for its criteria, investigators agree on definite definition for yes and the opposite for no in all questions. Variation was noticed between investigator's partial scores. Total average results were rated from ‘excellent’ to ‘very poor’ as described in previous studies.^30^

### HONcode

The HONcode is a quality certification that reflects the website's adherence to the eight ethical principles, including authorship, complementarity, privacy, attribution, justifiability, transparency, financial disclosure and advertising policy. Websites were evaluated for the presence of a HONcode seal which is awarded to websites that comply with the abovementioned criteria.^31^

### PIF TICK

PIF TICK is the only UK quality mark for trustworthy health information. To be awarded a PIF TICK mark seal, the organisation must pass through strong and professional processing of health information and meet the ten required criteria^28^ (Appendix C).

### BBB accreditation

BBB is an organisation that reflects customers’ reviews to create rating standards about the company. BBB accreditation is not applied for health information; it is awarded by a business that managed to make a good effort in resolving consumer complaints, and the seal displays the company's credibility.^32^ Table 4 in Appendix D presents the BBB standard criteria.^29^

## Results

Of the 17 websites included in our study, most of the websites are from the United States (n = 10, 58.8%), followed by the United Kingdom (n = 3, 17.6%), then Ireland (n = 2, 11.8%). Spain and Australia have the least presentation, each constituting n = 1 (5.9%) (Appendix E). This variation reflects ranking on Google search optimisation and English search.

Most included websites belong to charity affiliation category (n = 7, 41.2%), followed by academic and medical practice websites, each representing n = 3 (17.6%). Governmental and health portal have the least presentation, each constituting n = 2 (11.8%). US websites dominate its presence in most affiliation categories as follows: 100% of health portal, 66.6% of academic and 57.1% of charity websites (Appendix F).

Most of websites provide information on colorectal cancer immunotherapy (n = 13, 76.5%), while n = 4 (23.5%) give general cancer immunotherapy information, as presented in Table 7 in Appendix G. Websites presenting colorectal cancer information on immune checkpoint inhibitors constitute the majority (n = 9, 52.9%), indicating its recent approval by the Food and Drug Administration for treating microsatellite instability-high colorectal cancer.^3^ Fewer websites were noticed to present colorectal cancer immunotherapy information on cancer vaccines, as they are in clinical testing.^3^ From the overall colorectal cancer information on immune checkpoint inhibitors (n = 9, 52.9%), n = 2 (22.2%) is presented as press news, while n = 1 (11.1%) is presented as an interesting blog created by oncology consultant colorectal cancer patient.

As shown in Table 2, most of the websites have a score of <3 points on JAMA (n = 10, 58.8%) and the majority score (n = 7, 41.2%) 1 point. Minority of the websites score ≥3 points (n = 7, 41.2%) and almost more than half of them (n = 4, 23.5%) score 4 points. Excellent scoring on DISCERN is awarded by only two websites (11.8%), while 23.5% have a good score. Over half of websites (64.7%) scored fair/poor.

**Table 2.** Categorisation of websites according to JAMA score, mean DISCERN score and website's affiliation category.

JAMA score (range = 0–4) ^26^	Number of websites n (%) (Total n = 17)	Number of websites by affiliation category for each JAMA score n (Total n = 17)
4	4 (23.5)	Charity n = 2, medical practice n = 2
3	3 (17.6)	Charity n = 2, academic n = 1
2	3 (17.6)	Governmental n = 2, academic n = 1
1	7 (41.2)	Charity n = 3, medical practice n = 3, academic n = 1
0	0 (0)	N = 0
≥3	7 (41.2)	Charity n = 4, medical practice n = 2, academic n = 1
<3	10 (58.8)	Governmental n = 2, academic n = 2, charity n = 3, medical practice n = 3

DISCERN score (range = 16–80) ^30^	Number of websites n (%) (Total n = 17)	Number of websites by affiliation category for each DISCERN score category (Total n = 17)
63–80 (excellent)	2 (11.8)	Charity n = 1, health portal n = 1
51–62 (good)	4 (23.5)	Charity n = 3, medical practice n = 1
39–50 (fair)	7 (41.2)	Charity n = 2, academic n = 2, governmental n = 1, health portal n = 1, medical practice n = 1
27–38 (poor)	4 (23.5)	Medical practice n = 1, charity n = 1, academic n = 1, governmental n = 1
16–26 (very poor)	0 (0)	N = 0

Most of the websites with a score of ≥3 points on JAMA or excellent/good on DISCERN belong to charity affiliation, n = 4 for each (Table 2). Moreover, there is an overlap of three charity websites meeting the abovementioned criteria; thus, the charity affiliation category dominates its presence on websites fulfilling ≥3 points and excellent/good on JAMA and DISCERN, respectively (Table 3). All governmental and medical practice and most of the academic websites have a score of <3 points on JAMA, while all governmental and academic and most medical practice have fair/poor DISCERN scores (Table 2). All websites with a governmental affiliation category score <3 points and fair/poor on JAMA and DISCERN, respectively (Table 3).

**Table 3.** For the 17 websites, information on country, affiliation category, information presentation, total JAMA benchmark score, mean DISCERN score and presence/absence of other accreditations in descending DISCERN score order is presented.

Website link	Country	Affiliation category	Scope of the information provided	Treatment description	Authorship	Date currency	Total JAMA score	Mean DISCERN score	Other accreditations
1. https://www.macmillan.org.uk/cancer-information-and-support/treatments-and-drugs/targeted-therapies-for-bowel-cancer ^42^ Accessed on 20 July 2021	United Kingdom	Charity	Colorectal cancer immunotherapy	All treatments (immune checkpoint inhibitors, monoclonal antibodies, cancer vaccine, immune system modulators and cancer vaccine)	Authorised by the website team, reviewed by health professionals and approved by professor consultant oncologist	Last review date was in 2020, next review date is in 2023 and date on sources provided/no date of copyright	4	67 (excellent)	PIF TICK criteria
2. https://www.healthline.com/health/colorectal-cancer/colon-cancer-immunotherapy-faqs ^43^ Accessed on 20 July 2021	United States	Health portal	Colorectal cancer immunotherapy	Immune checkpoint inhibitors	One author and one medical reviewer provided; reviewer's credential is registered nurse	Last review date was in 2021, date of copyright was in 2021 and date on sources provided	4	64 (excellent)	HONcode certified
3. https://www.cancer.org/cancer/colon-rectal-cancer/treating/immunotherapy.html ^44^ Accessed on 21 July 2021	United States	Charity	Colorectal cancer immunotherapy	Immune checkpoint inhibitors	Authorised by website editorial team including doctors and nurses with oncology certifications	Last review date was in 2020 and date on sources provided/no date of copyright	4	58 (good)	None
4. https://www.cancer.ie/cancer-information-and-support/cancer-information/cancer-treatments-and-side-effects/immunotherapy ^45^ Accessed on 21 July 2021	Ireland	Charity	General immunotherapy	All treatments	No author	No dates	1	57 (good)	None
5. https://www.cancerresearch.org/en-us/immunotherapy/cancer-types/colorectal-cancer ^46^ Accessed on 21 July 2021	United States	Charity	Colorectal cancer immunotherapy	Monoclonal antibodies and immune checkpoint inhibitors	Reviewer name, affiliation and credential provided, no author name	Last review date (2020) and date on sources provided	3	52 (good)	BBB accreditation
6. https://www.therutherford.com/treatments/immunotherapy ^47^ Accessed on 22 July 2021	United Kingdom	Medical practice	General immunotherapy	All treatments with reference to Macmillan	No author	No date	1	51.5 (good)	None
7. https://www.cancer.net/blog/2018-02/what-you-need-know-about-immunotherapy-side-effects ^48^ Accessed on 22 July 2021	United States	Charity (blog)	Colorectal cancer immunotherapy	Checkpoint inhibitors	Author name and credential provided/no affiliation (author is consultant oncologist CRC patient)	Date of copyright only provided (2018)	3	47 (fair)	BBB-accredited charity
8. https://www.ccalliance.org/colorectal-cancer-information/treatments/immuno-oncology ^49^ Accessed on 22 July 2021	United States	Charity	Colorectal cancer immunotherapy	Monoclonal antibodies	No author	No date	1	47 (fair)	BBB-accredited charity
9. https://www.fda.gov/news-events/press-announcements/fda-approves-first-line-immunotherapy-patients-msi-hdmmr-metastatic-colorectal-cancer ^50^ Accessed 23 July 2021	United States	Government website	Colorectal cancer immunotherapy	Checkpoint inhibitors	No author	Date of publication only provided (2020)	2	45.5 (fair)	None
10. https://www.mdanderson.org/cancerwise/can-immunotherapy-treat-colorectal-cancer–where-we-are-and-what-is-ahead.h00-159459267.html ^5^ Accessed 23 July 2021	United States	Academic (university)	Colorectal cancer immunotherapy	All treatments	Only author name provided/no affiliation and credential	Only date of copyright provided (2021)	3	45 (fair)	None
11. 11. https://moffitt.org/cancers/colorectal-cancer/treatment/immunotherapy/ ^51^ Accessed 23 July 2021	United States	Medical practice	Colorectal cancer immunotherapy	Immune checkpoint inhibitors	No author	No date	1	43.5 (fair)	None
12. https://news.cancerconnect.com/colon-cancer/checkpoint-inhibitor-immunotherapy-delays-colorectal-cancer-progression^52^ Accessed on 24 July 2021	United States	Health portal	Colorectal cancer immunotherapy	Checkpoint inhibitors	Author name and credential only provided/no author affiliation	Date of copyright only provided (2020)	4	43 (fair)	None
13. https://www.uclh.nhs.uk/news/immunotherapy-bowel-cancer-could-change-clinical-practice#:∼:text=A%20large%20international%20trial%20involving,cancer%2C%20when%20compared%20with%20chemotherapy ^53^ Accessed 24 July 2021	United Kingdom	Academic (university)	Colorectal cancer immunotherapy	Checkpoint inhibitors	No author	Date of copyright only provided (2020)	2	40 (fair)	None
14. https://immucura.com/therapy ^54^ Accessed on 24 July 2021	Spain	Medical practice	General immunotherapy	Dendritic cell therapy vaccine	No author	No date	1	35 (poor)	None
15. https://www.bowelcanceraustralia.org/targeted-therapy-immunotherapy^55^ Accessed on 24 July 2021	Australia	Charity	Bowel cancer immunotherapy	Checkpoint inhibitors	No author	No dates	1	35 (poor)	None
16. 16. https://sarahcannon.com/for-patients/learn-about-cancer/colon-rectal-cancer/treatment.dot^56^ Accessed on 24 July 2021	United States	Academic (research institute)	Colorectal cancer immunotherapy	Cancer vaccine	No author	No dates	1	33.5 (poor)	None
17. https://www2.hse.ie/conditions/bowel-cancer/bowel-cancer-treatment.html ^57^ Accessed on 24 July 2021	Ireland	Government website	General immunotherapy	Monoclonal antibodies	No author	Last 2019 and next review 2022 date provided	2	31 (poor)	None

As shown in Table 1, all websites have full disclosure of ownership, sponsorship, advertising policy and sources of funding. A total of 58.8% of the websites present the date of copyright and/or the review date and provide up-to-date information. Overall, 41.2% of the websites have an author, while 29.4% wrote the sources of the information. Although one point is given for the presence of each criterion according to investigator's definitions, there is variation between websites in providing authorship, attribution of references and date of the information.

Table 3 presents all websites in descending order, with the highest quality score on DISCERN at the top. Total average DISCERN score ranges between 67 (highest) and 31(lowest). The top two websites according to the DISCERN evaluation meet all the JAMA criteria and have an additional accreditation presented as a PIF TICK or HONcode seal. Both provide information on colorectal cancer immunotherapy, and one is focusing on immune checkpoint inhibitors. The highest scoring is given to UK charity website with a PIF TICK mark seal. One HONcode seal is noted on included websites (5.8%).

As presented in Table 3, although good-quality websites show variation in total JAMA scoring between websites, the highest-scoring one on DISCERN meets all JAMA criteria. Most of the good-quality websites have a charity affiliation, and half of them give information on colorectal cancer immunotherapy.

Fair-quality websites are dominant, with the highest scoring given to two US charity organisations with BBB accreditation, as shown in Table 3. All of them provide information on colorectal cancer immunotherapy, with the majority focusing on immune checkpoint inhibitors. All BBB-accredited charity websites are from the United States and have good/poor DISCERN score.

Poor-quality websites on DISCERN have lower scoring on JAMA (<3) with no additional credentials, as presented in Table 3. They show variation in country and affiliation presentation. They are the only category to provide detailed information on cancer vaccine.

As in Table 3, a notable feature of higher-quality websites is their comprehensive presentation of authorship and date currency. They present both an author and a reviewer, with the website's ranked no. 1 information written, reviewed and approved by the website team, which includes healthcare professionals and professors in oncology. The no. 2 ranked website's information is written and reviewed by two different individuals with the reviewer credentials given as a registered nurse. Both websites provide a last review date and date on sources. However, no.1 is distinguished by writing the next review date while no. 2 the date of copyright.

The following results are presented in Table 4. On applying the DISCERN questionnaire to the websites, gaps were noticed in providing the following: the prognosis of patients if no treatment is given (n = 0, 0%), clear aim (n = 4, 23.5%) and the effect of treatments on patient’s quality of life (n = 7, 41.2%). All websites succeed to provide relevant information and describe the mechanism of action and benefits of treatments (n = 17, 100%). Most of the websites provide balanced information (n = 16, 94.1%), refer to areas of knowledge uncertainty (n = 15, 88.2%) and rated high to moderate on question 16 (n = 13, 76.4%). Small variation was noticed between websites in describing the following: more than one treatment choice (n = 12, 70.5%), support shared decision-making (n = 11, 64.7%), treatment risks (n = 11, 64.6%), details of additional sources of information (n = 11, 64.7%) and date on the information used or reported in the website (n = 10, 58.8%).

**Table 4.** DISCERN plus instrument (modified according to Borgmann et al. (2019)) questions, investigator's definition for full score and number of websites with yes, partial and no score.

Question number	What is investigated^58^	Investigators definition for the full score^58^	Number of websites n (%) with yes score (n = 17)	Number of websites n (%) with partial score (n = 17)	Number of websites n (%) with no score (n = 17)
1	Are the aims clear?	Clear aim with scope and audience is written.	0 (0)	4 (23.5)	13 (76.5)
2	Does it achieve its aims?	All the information expected from description of the aims has been provided.	0 (0)	4 (23.5)	0 (0)
3	Is it relevant?	Information about a treatment choice/s is relevant to user's need.	15 (88.2)	2 (11.8)	0 (0)
4	Is it clear what sources of information were used to compile the publication (other than the author or producer)?	The main statements made about treatment choices are accompanied by a reference to the sources associated with means of checking the sources used, such as a bibliography or external links to the online sources.	0 (0)	9 (52.9)	8 (47.1)
5	Is it clear when the information used or reported in the publication was produced?	Dates of the main sources of information used to compile the publication with the revision dates (not reprinting date) and the copyright date are provided.	4 (23.5)	6 (35.3)	7 (41.2)
6	Is it balanced and unbiased?	The publication is written from an objective point of view, or a range of sources of information was used to compile the publication or evidence of an external assessment of the publication.	5 (29.4)	11 (64.7)	1 (5.9)
7	Does it provide details of additional sources of support and information?	The publication provides full details of any additional source other than local branches of the same organisation. Bibliography is considered an additional source.	5 (29.4)	6 (35.3)	6 (35.3)
8	Does it refer to areas of uncertainty?	The publication includes a clear reference to any uncertainty regarding treatment choices, such as gaps in knowledge or differences in expert opinion concerning treatment choices or if a treatment choice affects everyone in the same way.	7 (41.2)	8 (47.1)	2 (11.8)
9	Does it describe how each treatment works?	The description of each treatment includes details of how it works, route of administration and how often it is given.	4 (23.5)	13 (76.5)	0 (0)
10	Does it describe the benefits of each treatment?	A benefit is described for each treatment, which includes controlling the symptoms, preventing recurrence of the condition and eliminating the condition, either short-term or long-term.	17 (100)	0 (0)	0 (0)
11	Does it describe the risks of each treatment?	A risk is described for each treatment, which includes side effects, complications and adverse reactions to treatments, both short-term and long-term.	8 (47)	3 (17.6)	6 (35.3)
12	Does it describe what would happen if no treatment is used?	There is a clear description of a risk or a benefit associated with any no treatment option.	0 (0)	0 (0)	17 (100)
13	Does it describe how the treatment choices affect overall quality of life?	Description of the effects of the treatment choices on day-to-day activity or relationships with family, friends and carers.	0 (0)	7 (41.2)	10 (58.8)
14	Is it clear that there may be more than one possible treatment choice?	The publication makes it very clear that there may be more than one possible treatment choices.	9 (52.9)	3 (17.6)	5 (29.4)
**15**	Does it provide support for shared decision-making?	The publication provides very good support for shared decision-making, that is, suggestions of things to discuss with family, friends, doctors or other health professionals concerning treatment choices.	8 (47.1)	3 (17.6)	6 (35.3)
**16**	Based on the answers to all of the above questions, rate the overall quality of the publication as a source of information about treatment choices.	The publication rated high (4 or above) on most questions.	3 (17.6)	10 (58.8)	4 (23.5)

As presented in Table 5 in Appendix H, the website ranked no. 3 on DISCERN shows the earliest presentation on Google search in all search terms, followed by no. 2 ranked website. The website ranked no. 1 appears on the first two search terms only and shows its latest presentation among others. The first search term ‘colon cancer immunotherapy’ has the highest exclusion and inclusion rate with no duplicates (Table 1 in Appendix A). It also shows the earliest appearance of the top three websites (10th, 6th, and 3rd) on the first page (first 10 hits), although in inverse order (Table 5 in Appendix H). Moreover, all the top 3 websites provide information specifically on colorectal cancer immunotherapy (Table 4). So, the first search term can be recommended as the best for patients seeking information on colorectal cancer immunotherapy.

Figure 2 in Appendix J presents a mind map for problem definition in our investigation.

## Discussion

We found the majority of websites describing colorectal cancer immunotherapy were of low to moderate quality using the JAMA and fair/poor on DISCERN quality tools. A substantial variation in the information provided to patients on treatment effectiveness and impact of treatment on quality of life, authorship details and dating of information was noted. Additional accreditations such as a PIF TICK and HONcode seal were included in two websites. This study identifies gaps in online treatment information and missing details that denote a high quality of information.

A major finding of our study was that most of the websites scored <3 points on JAMA and fair/poor on DISCERN. This finding was also reported in studies done by Kaicker and Dang and Olkun and Demirkaya (2016, 2018).^27,30^ However, this doesn’t support the previous research done in the United States where Google returns higher reliability scores than Yahoo and Bing and surprisingly MedlinePlus.^18^ Janssen et al. (2019)^33^ study also confirms good-quality information about prostate cancer radiotherapy on the internet.

In our investigation, most of charity websites score ≥3 points and excellent/good on JAMA and DISCERN, respectively, indicating their higher reliability, in accordance with a study done by Janssen et al. (2019).^33^

All governmental and most academic and medical practice websites were ranked fair/poor on DISCERN. This result is supported by Janssen et al.’s^33^ (2019) study and disagrees with Corcelles et al.’s^34^ (2015) research findings, which reported the commercial websites as the worst quality and the academic as the best.

Gaps were noticed in providing authorship, sources and date on website's information. These results match those observed in earlier studies done in Turkey and Canada (2018, 2016).^27,30^

Most of the websites fail to mention clear aim; however, this has not previously been described in studies done in India and Canada (2015, 2016), where websites scored well in the categories of providing appropriate aim.^27,31^ Failure to describe the effects of treatments on patient's prognosis and quality of life was noticed in our investigation. These patterns were also seen in previous studies done by Kiran et al. and Kaicker and Dang (2015, 2016).^27,31^

The top two excellent websites on DISCERN meet all the JAMA criteria and have an additional accreditation. This observation was noted in a study done in Canada (2010)^35^ which reported higher DISCERN score in websites with seals of approval and UK (2015) study where HONcode seals are present in websites with high JAMA score.^18^ This outcome is contrary to that of Meric et al.^36^ and Laversin et al.^37^ who found none of the sites with a seal complied with all four JAMA benchmark criteria and 70% of websites with a seal did not meet the standards for certification, respectively.

Few HONcode seals were noted in our study and reported in studies done by Borgmann et al.^38^ on prostate cancer and radical prostatectomy, Alkhateeb’s^39^ group in prostate cancer and surgery study,^39^ and Janssen et al.^33^ in a study of prostate cancer and radiotherapy. These findings indicate lower HONcode certification frequency when the search is more focused.

Websites of excellent quality on DISCERN instrument were not top ranked by Google in any of the search terms used. This finding is consistent with a study done in Canada which reported higher-quality websites as not appearing on higher hits^27^ and disagrees with a study done by Tan et al.^40^ in a review of mesothelioma information on world wide web.

### Implications for practice

Based on our findings, attributing website's information to authors whose background is clearly described and adding date of information would be simple steps to quickly improve the quality of information about cancer treatments online. Writing an aim with the scope and target audiences and the effect of treatments on prognosis and quality of life will dramatically improve audience convenience and accountability.

The infodemiology approach in this study is useful to describe the deficits with online information. However, there are numerous opportunities to improve the usefulness and quality of online information. Using the incognito window with clearance of browser history can be helpful for internet consumers to reduce the effect of personalised search algorithms. Increasing public awareness of the pitfalls with online information and how to critically analyse online information is necessary. Notwithstanding, seeking the views of people using the websites and collaborating with them can be a useful step in future research to improve reliability of online information. Moreover, the recommendation of high-quality resources for patients by healthcare providers can help to improve timely self-empowerment of patients with the accurate cancer information.

### Strength and limitations

This investigation is the first to evaluate the quality of colorectal cancer immunotherapy information on websites using the DISCERN and JAMA tools combined with design thinking technique. We selected an unfamiliar treatment for most patients and oncology providers.^23^ The use of various quality stamps like HONcode and PIF TICK in the study is a strength. There are some limitations with this study. Only English websites are included. The internet is a dynamic process, and Google, as the only search engine used, is constantly updating its website ranking algorithm.^33^ It was not possible to include all patient websites for colorectal cancer, so high-quality websites may not be included in the assessment if they are not ranked in the top 20 by Google. There is an overlap between some of the affiliation categories used for websites, although they are done based on previous investigations.^18,25^ The limitation of using JAMA in our investigation is that simply perfect scoring is obtained by the presence of each criterion. As such, it is better used to measure the transparency of the websites as described in a study done by Silberg et al.^41^ that first described the JAMA score.

## Conclusion

Most of the websites discussing colorectal cancer immunotherapy are of low to moderate quality, so patients should be cautious about trusting the information provided on websites. Digital literacy training would be beneficial for improving lay audience identification of trustworthy information. The websites operated by charity organisations were of superior quality compared to hospital and sponsored medical news sites. Thus, non-commercial sites should be preferentially recommended to patients seeking online information.

## Supplemental Material

sj-docx-1-dhj-10.1177_20552076231205286 - Supplemental material for An infodemiology study on exploring the quality and reliability of colorectal cancer immunotherapy information Click here for additional data file. Supplemental material, sj-docx-1-dhj-10.1177_20552076231205286 for An infodemiology study on exploring the quality and reliability of colorectal cancer immunotherapy information by Hind Mohamed, Laura O’Malley and Dervla Kelly in DIGITAL HEALTH
