# Supplementary material for: An infodemiology study on exploring the quality and reliability of colorectal cancer immunotherapy information
Source: Digit Health. 2023 Oct 4;9:20552076231205286. doi: 10.1177/20552076231205286 (PMC10552482; doi:10.1177/20552076231205286)
Supplement: sj-docx-1-dhj-10.1177_20552076231205286 - Supplemental material for An infodemiology study on exploring the quality and reliability of colorectal cancer immunotherapy information [file sj-docx-1-dhj-10.1177_20552076231205286.docx]

**Supporting information:**

**Appendix A:**

**Table 1: Number of websites included, excluded, total duplicates and total relevant websites identified for each search term.**

| **Search term** | **Total No. of relevant (English) websites identified** | **Total No. of non-duplicates** | | **Total No. of duplicates** |
| --- | --- | --- | --- | --- |
|  |  | **No. of included websites** | **No. of excluded websites** |  |
| “Colorectal cancer immunotherapy” | 20 | 8 | 12 | 0 |
| “Colorectal cancer immunotherapy treatment” | 20 | 1 | 4 | 15 |
| “Colon cancer side effects” | 20 | 5 | 2 | 13 |
| “Bowel cancer outcomes” | 20 | 3 | 2 | 15 |
| Total websites | 80 | 17 | 20 | 43 |

**Appendix B:**

**Table 2: Results of excluded websites.**

| Search terms | No. of Journal websites | NO. Of biopharmaceutical company websites | No. of Scientific research websites | No. of Non- relevant websites | Total excluded websites per search term |
| --- | --- | --- | --- | --- | --- |
| “Colorectal cancer immunotherapy” | 8 | 2 | 1 | 1(other cancer type) | 12 |
| “Colorectal cancer immunotherapy treatment” | 1 | 2 | 1 | 0 | 4 |
| “Colon cancer side effects” | 0 | 0 | 1 | 1 (Limited information) | 2 |
| “Bowel cancer outcomes” | 0 | 2 | 0 | 0 | 2 |
| Total No. of excluded websites for all search terms | 9 | 6 | 3 | 2 | 20 |

**Appendix C:**

**Table 3: PIF TICK criteria description**

| **Criteria Number**  (28) | **Criteria description** (28) |
| --- | --- |
| **1** | Information is created using a consistent and documented process. |
| **2** | Staff are trained and supported to produce high-quality information. |
| **3** | Information meets an identified consumer need |
| **4** | Information is based on reliable, up-to-date evidence |
| **5** | Patients are involved in the development of health information |
| **6** | Information is written in plain English |
| **7** | Print and digital information is easy to use and navigate |
| **8** | Users can give feedback on information |
| **9** | Information is promoted to make sure it reaches those who need it |
| **10** | The impact of information is measured |

**Appendix D: Table 4: BBB charity accountability standards**

| Category (29) | Standards (29) |
| --- | --- |
| Governance | **1. Board Oversight** |
|  | **2. Board Size** |
|  | **3. Board Meetings** |
|  | **4. Board Compensation** |
|  | **5. Conflict of Interest** |
| Measuring Effectiveness | **6. Effectiveness Policy** |
|  | **7. Effectiveness Report** |
| Finances | **8. Program Expenses** |
|  | **9. Fund Raising Expenses** |
|  | **10. Accumulating Funds** |
|  | **11. Audit Report** |
|  | **12. Detailed Expense Breakdown** |
|  | **13. Accurate Expense Reporting** |
|  | **14. Budget Plan** |
| Fundraising & Information | **15. Accurate Materials** |
|  | **16. Annual Report** |
|  | **17. Website Disclosures** |
|  | **18. Donor Privacy** |
|  | **19. Cause Marketing Disclosures** |
|  | **20. Complaints** |

**Appendix E:**

**Table 5: Description of websites by country.**

| **Country** | **No. of websites n (%)** |
| --- | --- |
| US | 10 (58.8) |
| UK | 3 (17.6) |
| Ireland | 2 (11.8) |
| Spain | 1 (5.9) |
| Australia | 1 (5.9) |

**Appendix F:**

**Table 6: Description of websites by affiliation category and country.**

| **Affiliation category** | **Description** | **No. of websites n (%) by affiliation category** | **No. of websites n (%) by country** |
| --- | --- | --- | --- |
| Charity (18) | A website which is designed for a pure charity purpose and not associated with financial benefits (Chumber et al., 2015) . | 7 (41.2) | US n=4 (23.5),  UK n=1(5.9),  Ireland n=1(5.9),  Australia n=1(5.9) |
| Academic (25) | A website that belongs to an institution with a clear educational purpose, such as university hospitals and research institutes (Shen et al., 2021) | 3 (17.6)  [university websites 2 (11.8) and  research institute 1 (5.9)]. | US n=2 (11.8)  UK n=1 (5.8) |
| Medical practise (25) | Local hospital or oncological practise without an academic affiliation (Shen et al., 2021) | 3 (17.6) | US n=1 (5.9),  UK n=1 (5.9)  Spain n=1 (5.9) |
| Governmental (25) | Websites that end in .gov or maintained by national government (Shen et al., 2021) | 2 (11.8) | US n=1 (5.9),  Ireland n=1 (5.9) |
| Health portal (18) | Website with a main purpose of providing health and medical information (Chumber et al., 2015) | 2 (11.8) | US n=2 (11.8) |

**Appendix G:**

**Table 7: Description of websites by the scope of immunotherapy information and type of treatment discussed.**

| **The scope of immunotherapy information** | **No. of websites n (%)** | **Type of treatment discussed on the website** | **No. of websites**  **n (%)** |
| --- | --- | --- | --- |
| Colorectal cancer immunotherapy | 13 (76.5) | All treatments | 2 (11.8) |
|  |  | Immune checkpoint inhibitors | 9 (52.9)  [press news 2 (22.2), blog 1 (11.1)] |
|  |  | Monoclonal antibodies | 1 (5.9) |
|  |  | Cancer vaccine | 1 (5.9) |
| Cancer immunotherapy | 4 (23.5) | All treatments | 2 (11.8) |
|  |  | Dendritic cell therapy vaccine | 1 (5.9) |
|  |  | Monoclonal antibody | 1 (5.9) |

**Appendix H: Table 5: Google ranking of the top 3 websites for each search term.**

| **Top 3 websites in descending DISCERN score order** | **Google Ranking of the top 3 websites for each search term** | | | |
| --- | --- | --- | --- | --- |
|  | **“Colorectal cancer immunotherapy” term**  **(n=20)** | **“Colorectal cancer immunotherapy treatment” term (n=20)** | **“Colon cancer immunotherapy side effects” term (n=20)** | **“Bowel cancer immunotherapy outcomes” term (n=20)** |
| 1. https://www.macmillan.org.uk/cancer-information-and-support/treatments-and-drugs/targeted-therapies-for-bowel-cancer (42) Accessed on 20.7.2021 | 10th | 13th | Absent | Absent |
| 2. https://www.healthline.com/health/colorectal-cancer/colon-cancer-immunotherapy-faqs (43)  Accessed on 20.7.2021 | 6th | 11th | 13th | 12th |
| 3. https://www.cancer.org/cancer/colon-rectal-cancer/treating/immunotherapy.html (44)  Accessed on 21.7.2021 | 3rd | 8th | 5th | 7th |

**Appendix I:**

**Figure 1: Context mapping of literature review findings on patients concerns, barriers, gaps and needs about immunotherapy treatment**

| Concerns | Barriers | Gaps | Needs |
| --- | --- | --- | --- |
| -Impact of poor-quality  medical information on health  care decision (7).  -Inferior quality of care (7).  -Pain and fatigue (22)  -Finance (22)  -The future (22)  -Disruption of life (22)  -Being physically active (22) | -Access to high quality medical  information (7).  -Inequity in access to internet (7).  -Communication with healthcare  providers (23)  -Access to clinical trials (23)  -Access to hospice (23) | -Quality of life/ patient reported  outcomes (23)  -Post treatment complication rate (23)  -Survival and mortality rate (23)  -Shared decision making (23)  -Knowledge and understanding of  immunotherapy among oncology  providers (23) | -High reliable health information  sources (7)  -Patient safety (23)  -Person and family-centred care (23)  -Care coordination and communication (23)  -Appropriate treatment selection (23)  -Health equity (7). |

**Appendix J: Figure 2: Mind map presenting the study users, defined gaps, and recommended solutions. Figure 2: Consort flow diagram of website’s inclusion and exclusion for evaluation.**

**Caregivers**

**Colorectal cancer patients**

**Most colorectal cancer immunotherapy information on websites has low to moderate quality**

**Application of the quality tools criteria on writing website's information**

**Public**

**Users**

**Providing patients with high quality resources based on research findings**

**All governmental and most of academic and medical practice websites have fair to poor quality**

**Gaps**

**Exploring the quality and reliability of colorectal cancer immunotherapy information on patient-oriented websites**

**Lack of content on reporting the aim and effect of treatment on patient's prognosis and quality of life**

**Solutions**

**Increase patient and public awareness about application of quality tools on assessing online information.**

**Involvement of patients in colorectal immunotherapy research**

**Deficiency and variation on describing authors, sources and dates on the information provided**

**Design a smart innovative tool to check for website's reliability**
